# Synthesis, crystal structure and thermal properties of the dinuclear complex bis­(μ-4-methylpyridine *N*-oxide-κ^2^ *O*:*O*)bis­[(methanol-κ*O*)(4-methylpyridine *N*-oxide-κ*O*)bis­(thio­cyanato-κ*N*)cobalt(II)]

**DOI:** 10.1107/S2056989024003128

**Published:** 2024-04-18

**Authors:** Christian Näther, Inke Jess

**Affiliations:** a Institut für Anorganische Chemie, Universität Kiel, Germany; Tokyo University of Science, Japan

**Keywords:** synthesis, crystal structure, thermal properties, cobalt thio­cyanate coordination compound, 4-methyl­pyridine *N*-oxide

## Abstract

Reaction of Co(NCS)_2_ with 4-methyl­pyridine *N*-oxide in methanol leads to the formation of crystals of the title compound, [Co_2_(NCS)_4_(C_6_H_7_NO)_4_(CH_4_O)_2_] or Co_2_(NCS)_4_(4-methyl­pyridine *N*-oxide)_4_(methanol)_2_. The asymmetric unit consist of one Co^II^ cation, two thio­cyanate anions, two 4-methyl­pyridine *N*-oxide coligands and one methanol mol­ecule in general positions. The H atoms of one of the methyl groups are disordered and were refined using a split model. The Co^II^ cations octa­hedrally coordinate two terminal N-bonded thio­cyanate anions, three 4-methyl­pyridine *N*-oxide coligands and one methanol mol­ecule. Each two Co^II^ cations are linked by pairs of μ-1,1(*O*,*O*)-bridging 4-methyl­pyridine *N*-oxide coligands into dinuclear units that are located on centers of inversion. Powder X-ray diffraction (PXRD) investigations prove that the title compound is contaminated with a small amount of Co(NCS)_2_(4-meth­yl­pyridine *N*-oxide)_3_. Thermogravimetric investigations reveal that the methanol mol­ecules are removed in the beginning, leading to a compound with the composition Co(NCS)_2_(4-methyl­pyridine *N*-oxide), which has been reported in the literature and which is of poor crystallinity.

## Abstract 2

Reaction of Co(NCS)_2_ with 4-methyl­pyridine *N*-oxide in methanol leads to the formation of Co_2_(NCS)_4_(4-methyl­pyridine *N*-oxide)_4_(methanol)_2_, in which the Co^II^ cations are linked by pairs of μ-1,1-bridging 4-methyl­pyridine *N*-oxide coligands into centrosymmetric dinuclear units.

## 1. Chemical context

The synthesis of new coordination compounds and polymers is still an important topic in inorganic chemistry because of their versatile structural behavior and their varied physical properties. One important part of these investigations includes the synthesis of compounds with paramagnetic metal cations to prepare materials with promising magnetic behavior. In several cases, the cations are linked by small-sized anionic ligands and in this regard, compounds based on thio­cyanate anions are of inter­est because this anionic ligand can mediate magnetic exchange (Palion-Gazda *et al.*, 2015 ▸; Mekuimemba *et al.*, 2018 ▸; Shurdha *et al.*, 2013 ▸; Rams *et al.*, 2017 ▸, 2020 ▸). Compared to cyanides or azides, this anionic ligand shows many more coordination modes and consequently a more pronounced structural variability, leading to metal thio­cyanate substructures that consist of linear and corrugated chains or layered structures of different topology (Wöhlert *et al.*, 2013 ▸; Werner *et al.*, 2015 ▸; Neumann *et al.* 2018 ▸; Böhme *et al.*, 2020 ▸, 2022 ▸). However, most paramagnetic metal cations are not very chalcophilic and therefore, the N-terminal coordination mode frequently dominates over the various bridging modes.

However, in recent work we used pyridine *N*-oxide deriv­atives as coligands that can be terminally O-bonded or that can bridge two metal cations in the μ-1,1(*O*,*O*) bridging mode, leading to an enhanced structural variability. In the beginning, we focused on Co(NCS)_2_ compounds because, among other things, this cation is of special inter­est in terms of its magnetic properties (Murrie, 2010 ▸; Mautner *et al.*, 2018*a* ▸,*b* ▸; Rams *et al.*, 2017 ▸, 2020 ▸). In the course of this project, we became inter­ested in 4-methyl­pyridine *N*-oxide as a coligand. With this ligand, two compounds with the composition Co(NCS)_2_(4-methyl­pyridine *N*-oxide) (Refcode: MEQKOJ, Zhang *et al.*, 2006*a* ▸) and Co(NCS)_2_(4-methyl­pyridine *N*-oxide)(methanol) (Refcode: REKBUF; Shi *et al.*, 2006*a* ▸) have already been reported in the literature. In the first compound, the Co^II^ cations octa­hedrally coordinate two N- and two S-bonding thio­cyanate anions and two μ-1,1(*O*,*O*)-bridging 4-methyl­pyridine N-oxide coligands, and are connected by pairs of bridging thio­cyanate anions into corrugated chains. These chains are further linked into layers by μ-1,1(*O*,*O*)-bridging 4-methyl­pyridine *N*-oxide coligands (Zhang *et al.*, 2006*a* ▸). In the second compound, the Co^II^ cations sixfold coordinate two bridging and one terminal thio­cyanate anion, two O atoms of two bridging 4-methyl­pyridine *N*-oxide ligands and one methanol mol­ecule (Refcode: REKBUF; Shi *et al.*, 2006*a* ▸). The Co cations are linked by alternating pairs of μ-1,3-bridging thio­cyanate anions and μ-1,1(*O*,*O*)-bridging 4-methyl­pyridine *N*-oxide coligands into chains. 
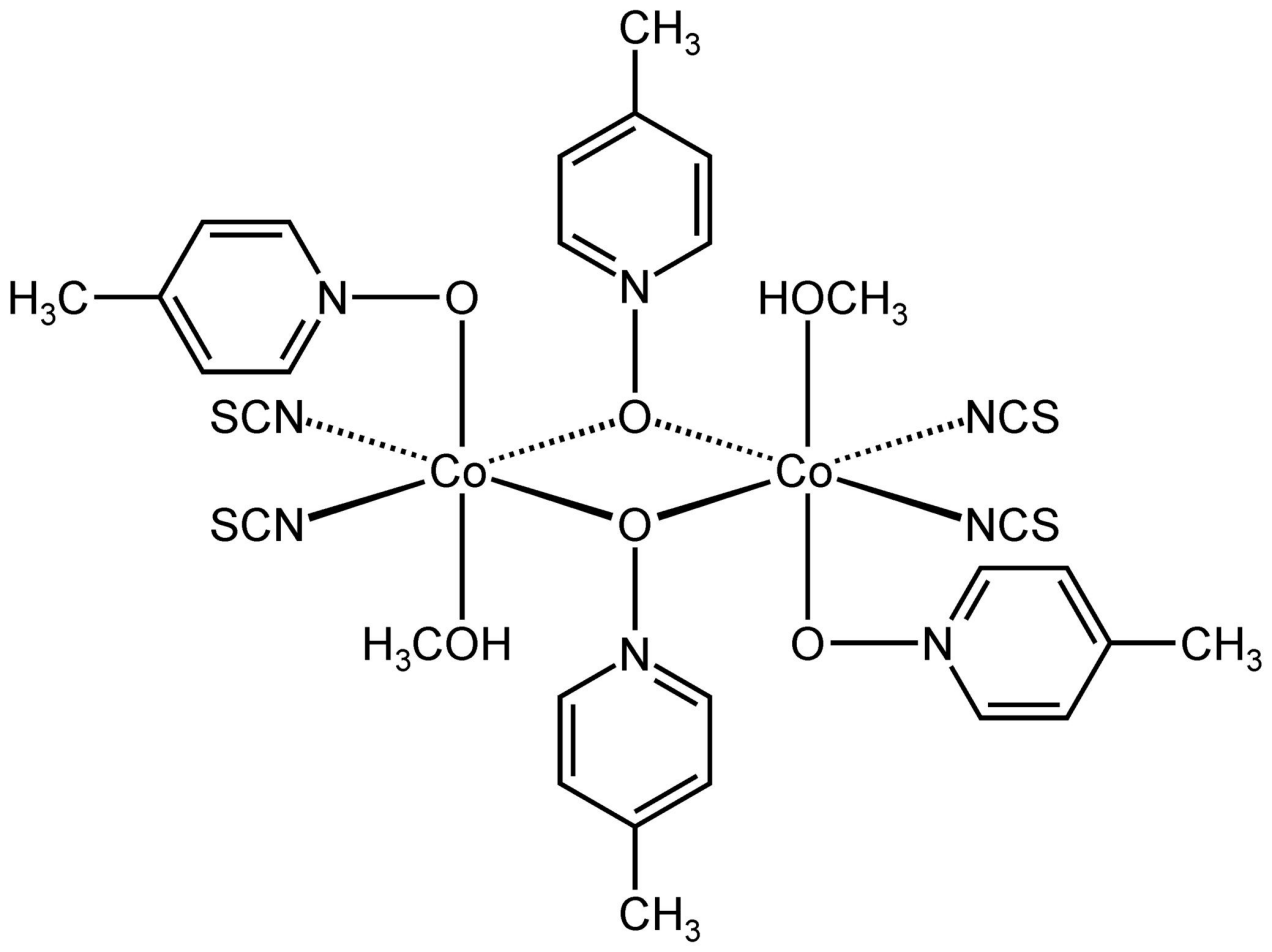


In our own synthetic work, we have added two additional compounds with the composition Co(NCS)_2_(4-methyl­pyridine *N*-oxide)_3_ and Co(NCS)_2_(4-methyl­pyridine *N*-oxide)_4_, that form discrete complexes with two different metal coordinations (Näther & Jess, 2024 ▸). In the latter compound, an octa­hedral coordination is observed, whereas the former shows a trigonal–bipyramidal coordination, which is relatively rare for Co^II^ cations. Surprisingly, this compound can easily be prepared, whereas only a few crystals of the complex with a sixfold coordination were accidentally obtained. Much effort was made to prepare Co(NCS)_2_(4-methyl­pyridine *N*-oxide)_4_ but without any success. In the course of these investigations, we always found additional reflections in some of the powder patterns of products prepared in methanol that do not correspond to the discrete complexes or to the coordination polymers mentioned above. Therefore, an additional crystalline phase based on Co(NCS)_2_ and 4-methyl­pyridine *N*-oxide must exist. Based on these findings the synthesis conditions were varied, leading to the formation of a new crystalline phase that was characterized by single-crystal X-ray diffraction. This proves that a dinuclear complex with methanol was obtained, that is somehow structurally related to Co(NCS)_2_(4-methyl­pyridine *N*-oxide)(methanol), which has already been reported in the literature (refcode REKBUF; Shi *et al.*, 2006*a* ▸).

## 2. Structural commentary

**Figure 1:**
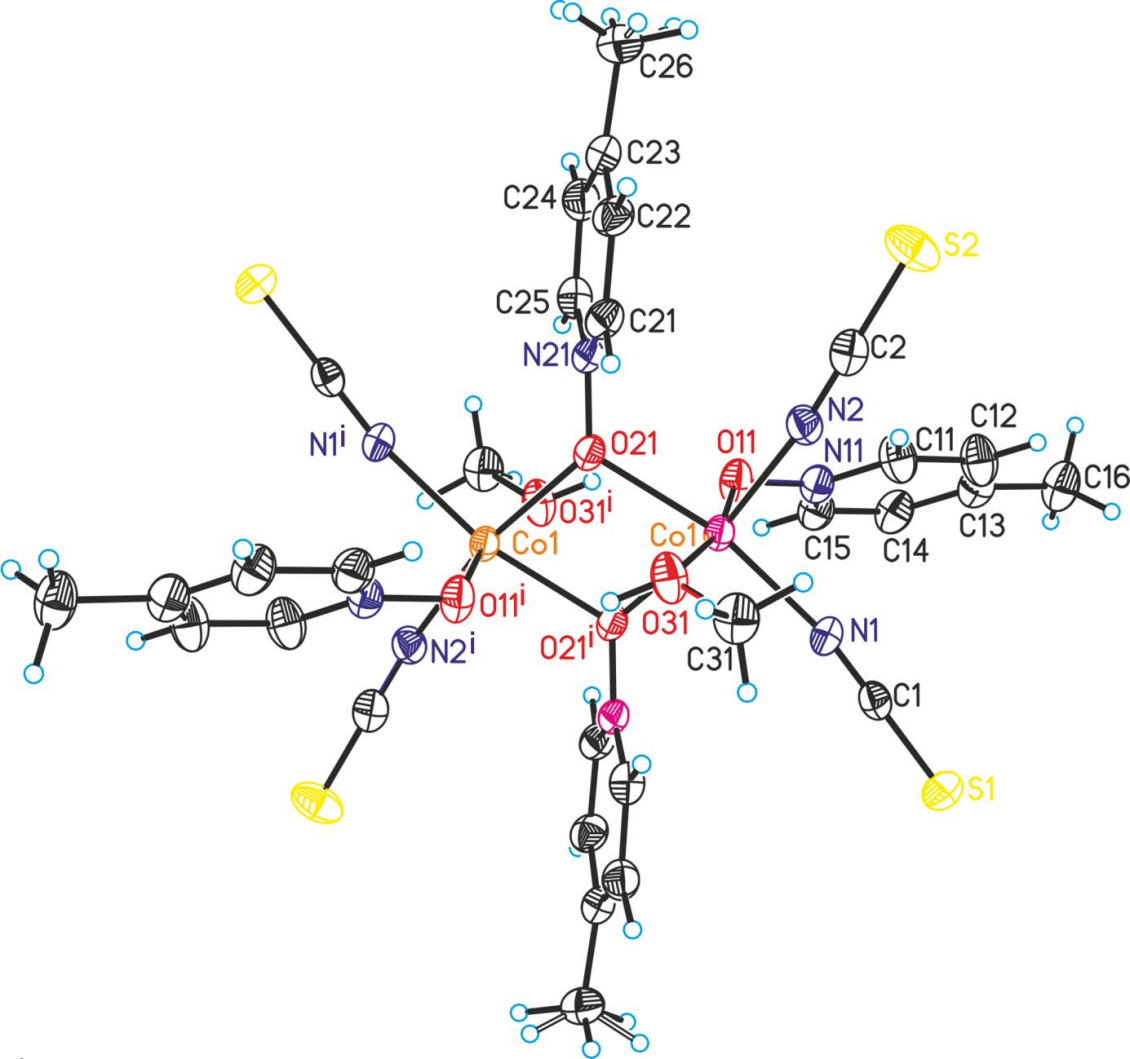
The molecular structure of the title compound with atom labelling and displacement ellipsoids drawn at the 50% probability level. The disorder of the H atoms of one of the methyl groups is shown with full and open bonds. [Symmetry code: (i) −*x* + 1, −*y*, −*z* + 1.]

The asymmetric unit of the title compound, Co_2_(NCS)_4_(4-methyl­pyridine *N*-oxide)_4_(methanol)_2_, consists of one cobalt cation, two thio­cyanate anions, one methanol mol­ecule and two 4-methyl­pyridine *N*-oxide coligands, all of them located in general positions. The Co cations sixfold coordinate two ter­minal N-bonding thio­cyanate anions, one methanol mol­ecule and one terminal as well as two μ-1,1(*O*,*O*)-bridging 4-methyl­pyridine *N*-oxide coligands (Fig. 1 ▸). Bond lengths and angles are similar to those in related compounds (Shi *et al.*, 2006*a* ▸) and show that the octa­hedra are slightly distorted (Table 1 ▸). Each two cobalt cations are linked *via* two μ-1,1(*O*,*O*)-bridging 4-methyl­pyridine *N*-oxide coligands into dinuclear units, with the Co_2_O_2_ rings that are the central motif located on centers of inversion (Fig. 1 ▸).

Similar Co_2_O_2_ rings are also observed in the related compound Co(NCS)_2_(4-methyl­pyridine *N*-oxide)(methanol), in which the Co cations are additionally linked *via* alternating pairs of μ-1,3-bridging thio­cyanate anions and μ-1,1(*O*,*O*)-bridging 4-methyl­pyridine *N*-oxide coligands into chains (Shi *et al.*, 2006*a* ▸).

## 3. Supra­molecular features

**Figure 2:**
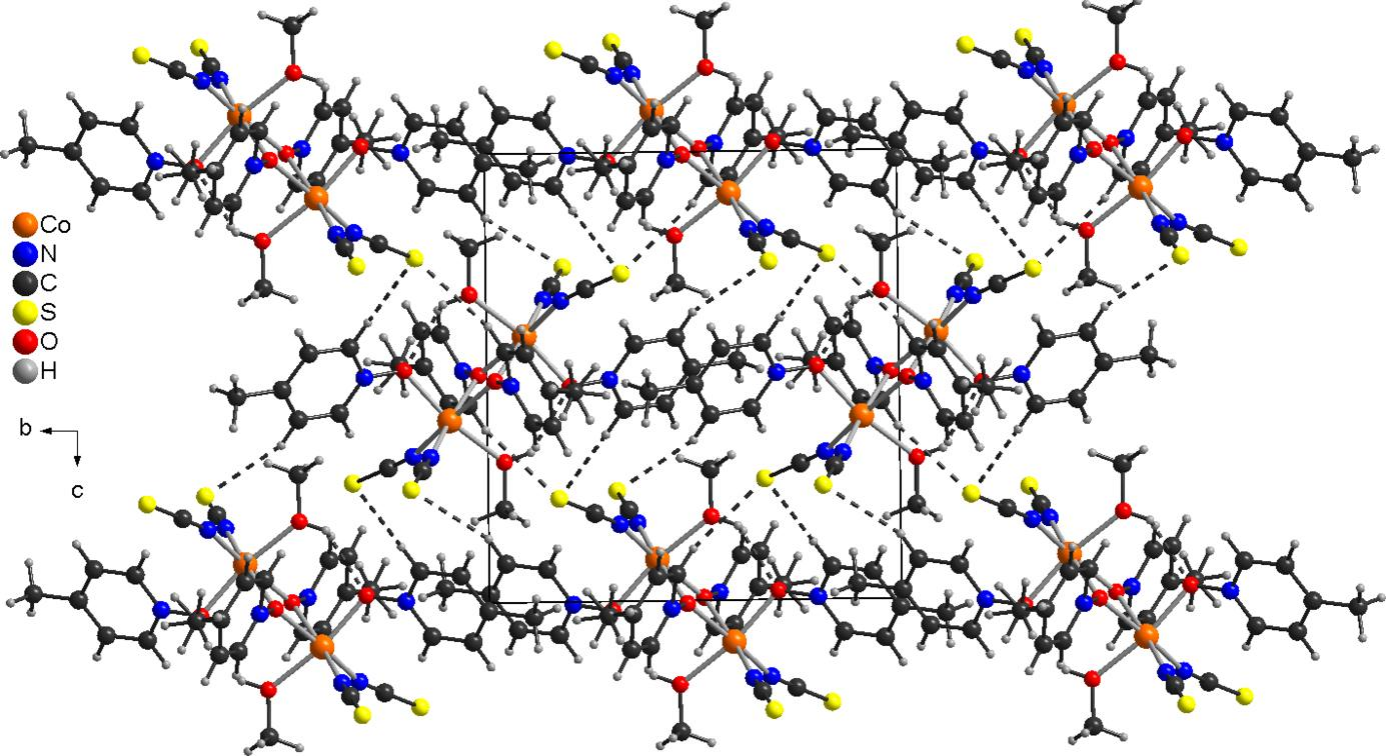
Crystal structure of the title compound in a view along the crystallographic *a* axis. Inter­molecular C—H⋯S and O—H⋯O hydrogen bonding is shown as dashed lines

In the crystal structure of the title compound, the dinculear units are arranged in columns along the crystallographic *a*-axis direction (Fig. 2 ▸). Several C—H⋯S, one C—H⋯O and one C—H⋯N contacts are observed between the complexes, but only for some of them are the C—H⋯*X* angles close to linearity and the H⋯*X* distances relatively short, indicating a significant inter­action (Fig. 2 ▸, Table 2 ▸).

## 4. Database survey

As mentioned above, two Co(NCS)_2_ compounds with 4-meth­yl­pyridine *N*-oxide are already reported in the Cambridge Structural Database (Version 5.43, last update March 2023; Groom *et al.*, 2016 ▸), including Co(NCS)_2_(4-methyl­pyridine *N*-oxide)(methanol) (CSD refcode REKBUF; Shi *et al.*, 2006*a* ▸) and Co(NCS)_2_(4-methyl­pyridine *N*-oxide) (refcode MEQKOJ; Zhang *et al.*, 2006*a* ▸). There are also two discrete complexes with the composition Co(NCS)_2_(4-methyl­pyridine *N*-oxide)_3_ and Co(NCS)_2_(4-methyl­pyridine *N*-oxide)_4_, as already mentioned in the *Chemical context* section (Näther & Jess, 2024 ▸).

With Ni^II^, a discrete complex with the composition Ni(NCS)_2_(4-methyl­pyridine *N*-oxide)_2_(H_2_O)_2_ has been reported that contains only terminally O-bonded coligands and which crystallizes as a monohydrate (Shi *et al.*, 2005*a* ▸). With Mn^II^, a similar discrete complex with the composition Mn(NCS)_2_(4-methyl­pyridine *N*-oxide)_2_(H_2_O)_2_ has also been reported (Mautner *et al.*, 2018*a* ▸,*b* ▸).

Two compounds with the composition *M*(NCS)_2_(4-methyl­pyridine *N*-oxide) (with *M* = Ni, Cd) are also found that are isotypic to its Co analog mentioned in the chemical context section [refcodes PEDSUN (Shi *et al.*, 2006*b* ▸), PEDSUN01 (Marsh, 2009 ▸) and TEQKAC (Shi *et al.*, 2006*c* ▸)].

With Cu(II), one compound with the composition Cu(NCS)_2_(4-methyl­pyridine *N*-oxide) is reported in which the Cu(II) cations are octa­hedrally coordinated by two N and three S-bonding thio­cyanate anions and one terminal O-coordinating 4-methyl­pyridine *N*-oxide) coligand (refcode TEB­TAW; Shi *et al.*, 2006*d* ▸). The Cu(II) cations are connected into linear chains by pairs of bridging thio­cyanate anions, that are further linked *via* Cu_2_S_2_ rings into double chains.

Finally, three isotypic compounds with the composition *M*(NCS)_2_)(acetato)_2_(H_2_O)_3_(4-methyl­pyridine *N*-oxide) (with *M* = Sm, Eu, Gd) are found [refcodes GIHBUV (Zhang & Shi, 2007 ▸) and PIJBIU and PIJBOA (Shi *et al.*, 2007*a* ▸)].

Some Co(NCS)_2_ compounds with other pyridine *N*-oxide derivatives are also known. This includes Co(NCS)_2_(pyridine *N*-oxide)_2_(H_2_O)_2_ and Co(NCS)_2_(3-hy­droxy­pyridine *N*-oxide)_2_(H_2_O)_2_ that consist of discrete octa­hedral complexes [refcodes FONBIU (Shi *et al.*, 2005*b* ▸) and IDOYEG (Shi *et al.*, 2006*e* ▸)]. This also includes Co(NCS)_2_(4-meth­oxy­pyridine *N*-oxide) that is isotypic to its 4-methyl­pyridine *N*-oxide analog (refcode TERRAK; Zhang *et al.*, 2006*b* ▸).

Finally, a compound with the composition Co(NCS)_2_(4-nitro­pyridine *N*-oxide) is also reported in the literature (refcode TILHIG; Shi *et al.*, 2007*b* ▸).

## 5. Additional investigations

**Figure 3:**
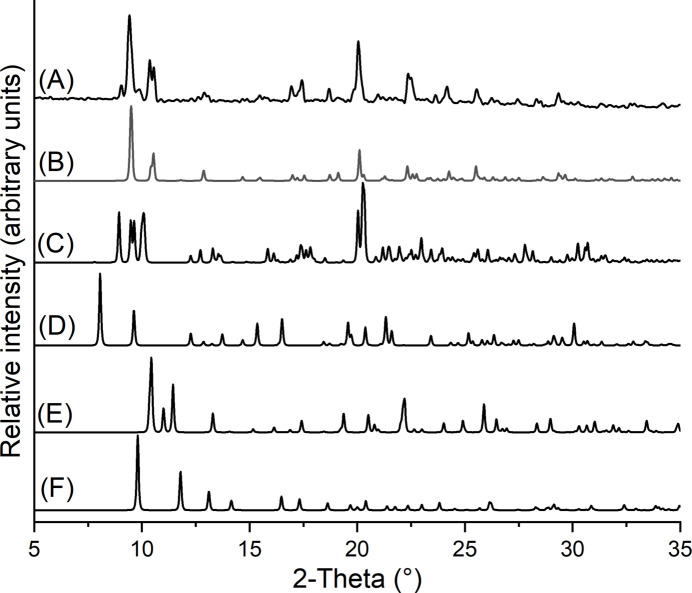
Experimental powder pattern of the title compound (A) together with the calculated pattern for the title compound (B), Co(NCS)_2_(4-methyl­pyridine *N*-oxide)_3_ (C, Näther & Jess, 2024 ▸), Co(NCS)_2_(4-methyl­pyridine *N*-oxide)_4_ (D, Näther & Jess, 2024 ▸), Co(NCS)_2_(4-methyl­pyridine *N*-oxide)(methanol) (E, Refcode: REKBUF; Shi *et al.*, 2006*a* ▸) and Co(NCS)_2_(4-methyl­pyridine *N*-oxide) (F, Refcode: MEQKOJ; Zhang *et al.*, 2006*a* ▸).

The title compound was also investigated by powder X-ray diffraction. Comparison of the experimental pattern with that calculated from single-crystal data reveals that this compound is of low crystallinity and that only a poor powder pattern can be obtained (Fig. 3 ▸). The low signal-to-noise ratio originates from the fact that only relatively large crystals were obtained, that could not be crushed into smaller crystals because in this case the compound started to decompose. However, it is obvious that no pure crystalline phase was obtained. In this context, it is noted that in those cases where different batches were investigated, the powder patterns always showed some differences. However, comparison of the experimental pattern with those calculated for the title compound and for Co(NCS)_2_(4-methyl­pyridine *N*-oxide) compounds retrieved from the literature indicate that the title compound is contaminated with a small amount of the discrete complex Co(NCS)_2_(4-methyl­pyridine *N*-oxide)_3_ (Näther & Jess, 2024 ▸). In fact, this is difficult to prove because the powder pattern was measured at room temperature, whereas the patterns calculated for the literature compounds are based in part on structure determinations at lower temperatures.

**Figure 4:**
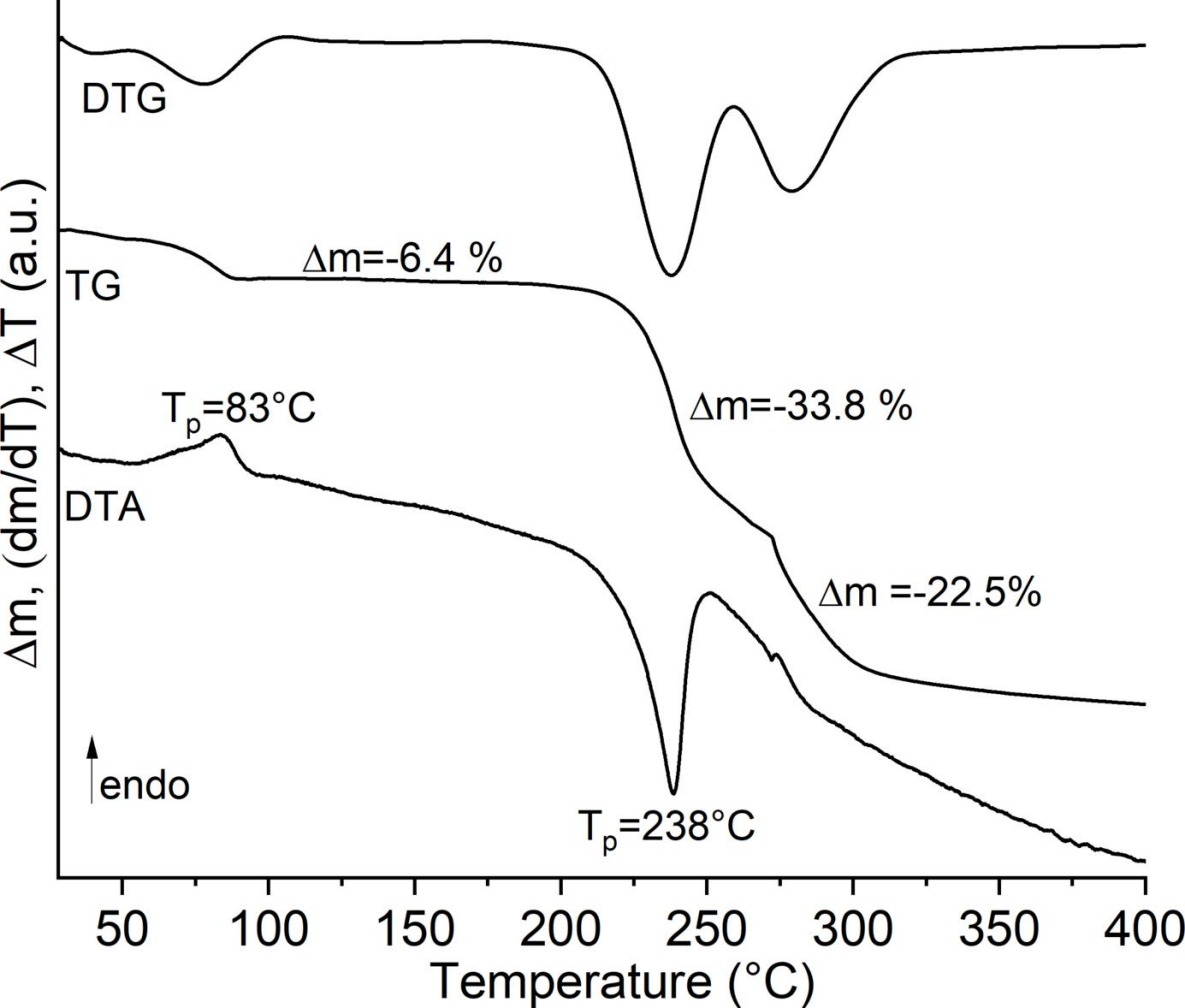
TG-DTA curve of the title compound measured at 8°C min^−1^.

However, measurements with thermogravimetry and differential thermoanalysis (TG-DTA) show three mass losses, of which the first is accompanied by an endothermic and the second by a strong exothermic signal in the DTA curve (Fig. 4 ▸). The first mass loss of 6.4% is a bit lower than that calculated for the removal of the methanol mol­ecules (7.5%), whereas the sum of the second and third mass losses is slightly higher than expected for the removal of all 4-methyl­pyridine *N*-oxide coligands (51.2%). However, the strong exothermic signal points to a decomposition of the coligands, as is usually observed for pyridine *N*-oxide derivatives (Näther & Jess, 2023 ▸, 2024 ▸). To characterize the compound formed after the first mass loss, it was isolated in a second TG run and investigated by PXRD. The powder pattern proves that a new crystalline phase of low crystallinity had been obtained that obviously contains a large amount of amorphous content (Figure S1). If the experimental pattern of the residue is compared with that calculated for Co(NCS)_2_(4-methyl­pyridine *N*-oxide) reported in the literature (Refcode: MEQKOJ, Zhang *et al.*, 2006*a* ▸), it is obvious that this compound has formed by methanol removal.

## 6. Synthesis and crystallization

Co(NCS)_2_ (99%) was purchased from Sigma Aldrich, 4-methyl­pyridine *N*-oxide (97%) from Thermo Scientific and methanol from Fisher Chemical.

**Synthesis:**

The title compound was prepared by the reaction of 0.5 mmol (87 mg) of Co(SCN)_2_ and 1 mmol (109 mg) of 4-methyl­pyridine *N* oxide in 1 mL of methanol. The reaction mixture was stored overnight, leading to the formation of violet-colored crystals that were always contaminated with Co(NCS)_2_(4-methyl­pyridine *N*-oxide)_3_ (Näther & Jess, 2024 ▸).

**Experimental details:**

The data collection for single-crystal structure analysis was performed using an XtaLAB Synergy, Dualflex, HyPix diffractometer from Rigaku with Cu *K*α radiation. The PXRD measurements were either performed with the single-crystal diffractometer mentioned above (Fig. S1) or with a Stoe Transmission Powder Diffraction System STADI P (Fig. 3 ▸) equipped with a MYTHEN 1K detector and a Johansson-type Ge(111) monochromator using Cu *K*α_1_ radiation (λ = 1.540598 Å). Thermogravimetry and differential thermoanalysis (TG-DTA) measurements were performed in a dynamic nitro­gen atmosphere in Al_2_O_3_ crucibles using a STA-PT 1000 thermobalance from Linseis. The instrument was calibrated using standard reference materials.

## 7. Refinement

Crystal data, data collection and structure refinement details are summarized in Table 3 ▸. The hydrogen atoms were positioned with idealized geometry and were refined with *U*_iso_(H) = 1.2*U*_eq_(C) (1.5 for methyl H atoms) using a riding model. The H atoms of one of the methyl groups are disordered and were refined using a split model with two orientations rotated to each other by 60°.

## Supplementary Material

Crystal structure: contains datablock(s) 1. DOI: 10.1107/S2056989024003128/jp2005sup1.cif

Figure S1. Experimental powder pattern of the residue obtained after methanol removal (top) and calculated pattern for Co(NCS)2(4-methylpyridine N-oxide retrieved from literature (Refcode: MEQKOJ, Zhang et al., 2006a). DOI: 10.1107/S2056989024003128/jp2005sup3.png

CCDC reference: 2347590

Additional supporting information:  crystallographic information; 3D view; checkCIF report

## Figures and Tables

**Table 1:** Selected geometric parameters (Å, °)

Co1—N1	2.0525 (18)	Co1—O21	2.1057 (15)
Co1—N2	2.0840 (18)	Co1—O21^i^	2.1043 (15)
Co1—O11	2.0543 (16)	Co1—O31	2.1301 (16)
			
N1—Co1—N2	96.79 (7)	O11—Co1—N2	96.57 (7)
N1—Co1—O11	96.07 (7)	O11—Co1—O21	83.57 (6)
N1—Co1—O21^i^	94.26 (7)	O11—Co1—O21^i^	87.62 (7)
N1—Co1—O21	166.79 (7)	O11—Co1—O31	167.80 (6)
N1—Co1—O31	95.14 (7)	O21^i^—Co1—O21	72.53 (6)
N2—Co1—O21^i^	167.69 (7)	O21^i^—Co1—O31	86.77 (7)
N2—Co1—O21	96.37 (7)	O21—Co1—O31	84.42 (7)
N2—Co1—O31	86.85 (7)		

Symmetry code: (i) 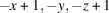 .

**Table 2:** Hydrogen-bond geometry (Å, °)

*D*—H⋯*A*	*D*—H	H⋯*A*	*D*⋯*A*	*D*—H⋯*A*
C11—H11⋯N2	0.95	2.40	3.225 (3)	145
C12—H12⋯S1^ii^	0.95	2.79	3.688 (3)	158
C15—H15⋯S2^iii^	0.95	2.68	3.609 (3)	167
C21—H21⋯S2^iv^	0.95	3.03	3.917 (2)	156
C22—H22⋯S1^v^	0.95	2.98	3.821 (2)	148
O31—H31⋯S1^vi^	0.84	2.97	3.6106 (18)	134
O31—H31⋯O11^i^	0.84	2.31	3.003 (2)	141
C31—H31*B*⋯S2^iv^	0.98	2.83	3.575 (3)	133

Symmetry codes: (i) 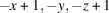 ; (ii) 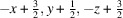 ; (iii) 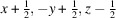 ; (iv) 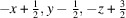 ; (v) 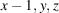 ; (vi) 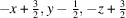 .

**Table 3:** Experimental details

Crystal data	
Chemical formula	[Co_2_(NCS)_4_(C_6_H_7_NO)_4_(CH_4_O)_2_]
*M*_r_	850.77
Crystal system, space group	Monoclinic, *P*2_1_/*n*
Temperature (K)	100
*a*, *b*, *c* (Å)	11.46665 (13), 12.37103 (15), 13.58185 (17)
β (°)	97.0894 (11)
*V* (Å^3^)	1911.91 (4)
*Z*	2
Radiation type	Cu *K*α
μ (mm^−1^)	9.27
Crystal size (mm)	0.21 × 0.14 × 0.1

Data collection	
Diffractometer	XtaLAB Synergy, Dualflex, HyPix
Absorption correction	Multi-scan (*CrysAlis PRO*; Rigaku OD, 2023 ▸)
*T*_min_, *T*_max_	0.529, 1.000
No. of measured, independent and observed [*I* > 2σ(*I*)] reflections	13370, 4111, 3945
*R*_int_	0.024
(sin θ/λ)_max_ (Å^−1^)	0.640

Refinement	
*R*[*F* ^2^ > 2σ(*F* ^2^)], *wR*(*F* ^2^), *S*	0.038, 0.103, 1.09
No. of reflections	4111
No. of parameters	231
H-atom treatment	H atoms treated by a mixture of independent and constrained refinement
Δρ_max_, Δρ_min_ (e Å^−3^)	0.67, −0.57

Computer programs: *CrysAlis PRO* (Rigaku OD, 2023 ▸), *SHELXT2014/5* (Sheldrick, 2015*a* ▸), *SHELXL2016/6* (Sheldrick, 2015*b* ▸), *DIAMOND* (Brandenburg & Putz, 1999 ▸), *XP* in *SHELXTL*-PC (Sheldrick, 2008 ▸) and *publCIF* (Westrip, 2010 ▸).

## supplementary crystallographic information

### Crystal data

**Table:**

[Co_2_(NCS)_4_(C_6_H_7_NO)_4_(CH_4_O)_2_]	*F*(000) = 876
*M**_r_* = 850.77	*D*_x_ = 1.478 Mg m^−^^3^
Monoclinic, *P*2_1_/*n*	Cu *K*α radiation, λ = 1.54184 Å
*a* = 11.46665 (13) Å	Cell parameters from 9687 reflections
*b* = 12.37103 (15) Å	θ = 4.8–80.1°
*c* = 13.58185 (17) Å	µ = 9.27 mm^−^^1^
β = 97.0894 (11)°	*T* = 100 K
*V* = 1911.91 (4) Å^3^	Block, violet
*Z* = 2	0.21 × 0.14 × 0.1 mm

### Data collection

**Table:**

XtaLAB Synergy, Dualflex, HyPix diffractometer	4111 independent reflections
Radiation source: micro-focus sealed X-ray tube, PhotonJet (Cu) X-ray Source	3945 reflections with *I* > 2σ(*I*)
Mirror monochromator	*R*_int_ = 0.024
Detector resolution: 10.0000 pixels mm^-1^	θ_max_ = 80.6°, θ_min_ = 4.8°
ω scans	*h* = −14→14
Absorption correction: multi-scan (CrysAlisPro; Rigaku OD, 2023)	*k* = −14→15
*T*_min_ = 0.529, *T*_max_ = 1.000	*l* = −16→17
13370 measured reflections	

### Refinement

**Table:**

Refinement on *F*^2^	Primary atom site location: dual
Least-squares matrix: full	Hydrogen site location: inferred from neighbouring sites
*R*[*F*^2^ > 2σ(*F*^2^)] = 0.038	H atoms treated by a mixture of independent and constrained refinement
*wR*(*F*^2^) = 0.103	*w* = 1/[σ^2^(*F*_o_^2^) + (0.0563*P*)^2^ + 1.4735*P*] where *P* = (*F*_o_^2^ + 2*F*_c_^2^)/3
*S* = 1.09	(Δ/σ)_max_ = 0.001
4111 reflections	Δρ_max_ = 0.67 e Å^−^^3^
231 parameters	Δρ_min_ = −0.57 e Å^−^^3^
0 restraints	

### Special details

**Table:**

Geometry. All esds (except the esd in the dihedral angle between two l.s. planes) are estimated using the full covariance matrix. The cell esds are taken into account individually in the estimation of esds in distances, angles and torsion angles; correlations between esds in cell parameters are only used when they are defined by crystal symmetry. An approximate (isotropic) treatment of cell esds is used for estimating esds involving l.s. planes.

### Fractional atomic coordinates and isotropic or equivalent isotropic displacement parameters (Å^2^)

**Table:**

	*x*	*y*	*z*	*U*_iso_*/*U*_eq_	Occ. (<1)
Co1	0.52596 (3)	0.09188 (3)	0.59292 (2)	0.02238 (11)	
N1	0.68049 (16)	0.13883 (15)	0.67419 (14)	0.0275 (4)	
C1	0.77744 (19)	0.15936 (17)	0.70365 (15)	0.0251 (4)	
S1	0.91377 (5)	0.18723 (5)	0.74606 (5)	0.03513 (15)	
N2	0.41432 (16)	0.18148 (15)	0.67148 (14)	0.0289 (4)	
C2	0.3463 (2)	0.24201 (18)	0.69672 (16)	0.0293 (4)	
S2	0.25069 (6)	0.32838 (5)	0.72994 (5)	0.04178 (17)	
O11	0.53273 (15)	0.20122 (12)	0.47994 (11)	0.0309 (3)	
N11	0.58635 (16)	0.29720 (15)	0.49406 (13)	0.0272 (4)	
C11	0.5698 (2)	0.3592 (2)	0.57280 (18)	0.0364 (5)	
H11	0.520567	0.334127	0.619345	0.044*	
C12	0.6237 (3)	0.4587 (2)	0.58635 (19)	0.0393 (6)	
H12	0.612097	0.501094	0.642635	0.047*	
C13	0.6944 (2)	0.4972 (2)	0.51892 (17)	0.0339 (5)	
C14	0.7098 (2)	0.4312 (2)	0.43895 (18)	0.0357 (5)	
H14	0.758612	0.454784	0.391503	0.043*	
C15	0.6552 (2)	0.3316 (2)	0.42736 (17)	0.0322 (5)	
H15	0.666465	0.287397	0.372102	0.039*	
C16	0.7506 (3)	0.6070 (2)	0.53342 (19)	0.0409 (6)	
H16A	0.814718	0.613082	0.492246	0.061*	
H16B	0.781897	0.616432	0.603361	0.061*	
H16C	0.691764	0.663039	0.514100	0.061*	
O21	0.39294 (13)	0.02026 (13)	0.49327 (12)	0.0287 (3)	
N21	0.28216 (15)	0.05826 (15)	0.48253 (13)	0.0242 (3)	
C21	0.21856 (19)	0.04578 (18)	0.55877 (17)	0.0283 (4)	
H21	0.248923	0.004642	0.615235	0.034*	
C22	0.1092 (2)	0.09302 (19)	0.55439 (18)	0.0310 (5)	
H22	0.064668	0.084973	0.608466	0.037*	
C23	0.0632 (2)	0.15256 (19)	0.47132 (18)	0.0316 (5)	
C24	0.1299 (2)	0.15751 (19)	0.39241 (18)	0.0325 (5)	
H24	0.099213	0.193206	0.332881	0.039*	
C25	0.2396 (2)	0.11137 (18)	0.39968 (17)	0.0297 (4)	
H25	0.285208	0.117040	0.346108	0.036*	
C26	−0.0518 (2)	0.2114 (2)	0.4683 (2)	0.0418 (6)	
H26A	−0.068918	0.248973	0.404593	0.063*	0.5
H26B	−0.046939	0.264182	0.522437	0.063*	0.5
H26C	−0.114632	0.159457	0.475782	0.063*	0.5
H26D	−0.084742	0.199435	0.530615	0.063*	0.5
H26E	−0.106720	0.184226	0.412771	0.063*	0.5
H26F	−0.039027	0.288951	0.459426	0.063*	0.5
O31	0.49549 (16)	−0.04034 (13)	0.68691 (12)	0.0340 (4)	
H31	0.495536	−0.104304	0.666149	0.061 (11)*	
C31	0.5284 (2)	−0.0392 (2)	0.79179 (18)	0.0373 (5)	
H31A	0.611351	−0.059417	0.806657	0.056*	
H31B	0.479797	−0.090838	0.823227	0.056*	
H31C	0.516711	0.033542	0.817470	0.056*	

### Atomic displacement parameters (Å^2^)

**Table:**

	*U*^11^	*U*^22^	*U*^33^	*U*^12^	*U*^13^	*U*^23^
Co1	0.02249 (18)	0.02112 (18)	0.02309 (18)	−0.00307 (12)	0.00103 (12)	−0.00346 (12)
N1	0.0264 (9)	0.0278 (9)	0.0269 (9)	−0.0040 (7)	−0.0024 (7)	−0.0050 (7)
C1	0.0333 (11)	0.0209 (9)	0.0213 (9)	0.0016 (8)	0.0046 (8)	−0.0018 (7)
S1	0.0250 (3)	0.0377 (3)	0.0412 (3)	−0.0034 (2)	−0.0017 (2)	−0.0031 (2)
N2	0.0284 (9)	0.0286 (9)	0.0302 (9)	0.0000 (7)	0.0059 (7)	−0.0062 (7)
C2	0.0368 (11)	0.0288 (11)	0.0222 (10)	−0.0063 (9)	0.0032 (8)	0.0025 (8)
S2	0.0547 (4)	0.0415 (3)	0.0316 (3)	0.0200 (3)	0.0152 (3)	0.0069 (2)
O11	0.0412 (9)	0.0245 (7)	0.0257 (7)	−0.0083 (6)	−0.0016 (6)	0.0000 (6)
N11	0.0324 (9)	0.0234 (9)	0.0245 (8)	−0.0032 (7)	−0.0013 (7)	0.0025 (7)
C11	0.0514 (14)	0.0278 (11)	0.0316 (12)	−0.0054 (10)	0.0113 (10)	−0.0004 (9)
C12	0.0575 (16)	0.0279 (12)	0.0338 (12)	−0.0083 (11)	0.0102 (11)	−0.0009 (9)
C13	0.0397 (12)	0.0307 (11)	0.0296 (11)	−0.0060 (10)	−0.0028 (9)	0.0058 (9)
C14	0.0378 (12)	0.0380 (12)	0.0308 (12)	−0.0095 (10)	0.0022 (10)	0.0034 (10)
C15	0.0339 (11)	0.0345 (12)	0.0275 (11)	−0.0022 (9)	0.0008 (9)	−0.0001 (9)
C16	0.0539 (16)	0.0353 (13)	0.0318 (12)	−0.0149 (11)	−0.0009 (11)	0.0051 (10)
O21	0.0207 (7)	0.0321 (8)	0.0322 (8)	−0.0013 (6)	−0.0005 (6)	−0.0131 (6)
N21	0.0197 (8)	0.0234 (8)	0.0291 (9)	−0.0031 (7)	0.0012 (6)	−0.0051 (7)
C21	0.0262 (10)	0.0288 (11)	0.0293 (10)	−0.0052 (8)	0.0010 (8)	0.0013 (8)
C22	0.0243 (10)	0.0362 (12)	0.0329 (12)	−0.0039 (8)	0.0052 (9)	−0.0026 (9)
C23	0.0273 (10)	0.0302 (11)	0.0358 (12)	−0.0021 (9)	−0.0026 (9)	−0.0072 (9)
C24	0.0369 (12)	0.0274 (11)	0.0317 (11)	−0.0010 (9)	−0.0012 (9)	−0.0008 (9)
C25	0.0330 (11)	0.0275 (10)	0.0288 (11)	−0.0044 (9)	0.0046 (9)	−0.0033 (9)
C26	0.0310 (12)	0.0459 (15)	0.0467 (15)	0.0076 (11)	−0.0025 (10)	−0.0067 (12)
O31	0.0465 (9)	0.0235 (8)	0.0328 (8)	−0.0070 (7)	0.0085 (7)	−0.0028 (6)
C31	0.0445 (13)	0.0345 (12)	0.0330 (12)	−0.0045 (10)	0.0053 (10)	0.0029 (10)

### Geometric parameters (Å, º)

**Table:**

Co1—N1	2.0525 (18)	C16—H16C	0.9800
Co1—N2	2.0840 (18)	O21—N21	1.345 (2)
Co1—O11	2.0543 (16)	N21—C21	1.347 (3)
Co1—O21	2.1057 (15)	N21—C25	1.342 (3)
Co1—O21^i^	2.1043 (15)	C21—H21	0.9500
Co1—O31	2.1301 (16)	C21—C22	1.378 (3)
N1—C1	1.162 (3)	C22—H22	0.9500
C1—S1	1.635 (2)	C22—C23	1.395 (3)
N2—C2	1.163 (3)	C23—C24	1.393 (3)
C2—S2	1.634 (2)	C23—C26	1.503 (3)
O11—N11	1.340 (2)	C24—H24	0.9500
N11—C11	1.348 (3)	C24—C25	1.373 (3)
N11—C15	1.342 (3)	C25—H25	0.9500
C11—H11	0.9500	C26—H26A	0.9800
C11—C12	1.379 (3)	C26—H26B	0.9800
C12—H12	0.9500	C26—H26C	0.9800
C12—C13	1.380 (3)	C26—H26D	0.9800
C13—C14	1.388 (4)	C26—H26E	0.9800
C13—C16	1.506 (3)	C26—H26F	0.9800
C14—H14	0.9500	O31—H31	0.8400
C14—C15	1.382 (3)	O31—C31	1.428 (3)
C15—H15	0.9500	C31—H31A	0.9800
C16—H16A	0.9800	C31—H31B	0.9800
C16—H16B	0.9800	C31—H31C	0.9800
			
N1—Co1—N2	96.79 (7)	C25—N21—O21	120.21 (18)
N1—Co1—O11	96.07 (7)	C25—N21—C21	121.67 (19)
N1—Co1—O21^i^	94.26 (7)	N21—C21—H21	120.2
N1—Co1—O21	166.79 (7)	N21—C21—C22	119.6 (2)
N1—Co1—O31	95.14 (7)	C22—C21—H21	120.2
N2—Co1—O21^i^	167.69 (7)	C21—C22—H22	119.7
N2—Co1—O21	96.37 (7)	C21—C22—C23	120.6 (2)
N2—Co1—O31	86.85 (7)	C23—C22—H22	119.7
O11—Co1—N2	96.57 (7)	C22—C23—C26	121.4 (2)
O11—Co1—O21	83.57 (6)	C24—C23—C22	117.2 (2)
O11—Co1—O21^i^	87.62 (7)	C24—C23—C26	121.4 (2)
O11—Co1—O31	167.80 (6)	C23—C24—H24	119.6
O21^i^—Co1—O21	72.53 (6)	C25—C24—C23	120.7 (2)
O21^i^—Co1—O31	86.77 (7)	C25—C24—H24	119.6
O21—Co1—O31	84.42 (7)	N21—C25—C24	119.9 (2)
C1—N1—Co1	166.57 (18)	N21—C25—H25	120.0
N1—C1—S1	179.4 (2)	C24—C25—H25	120.0
C2—N2—Co1	165.94 (18)	C23—C26—H26A	109.5
N2—C2—S2	178.8 (2)	C23—C26—H26B	109.5
N11—O11—Co1	122.38 (12)	C23—C26—H26C	109.5
O11—N11—C11	120.70 (19)	C23—C26—H26D	109.5
O11—N11—C15	118.81 (19)	C23—C26—H26E	109.5
C15—N11—C11	120.5 (2)	C23—C26—H26F	109.5
N11—C11—H11	119.7	H26A—C26—H26B	109.5
N11—C11—C12	120.6 (2)	H26A—C26—H26C	109.5
C12—C11—H11	119.7	H26A—C26—H26D	141.1
C11—C12—H12	119.7	H26A—C26—H26E	56.3
C11—C12—C13	120.6 (2)	H26A—C26—H26F	56.3
C13—C12—H12	119.7	H26B—C26—H26C	109.5
C12—C13—C14	117.3 (2)	H26B—C26—H26D	56.3
C12—C13—C16	120.1 (2)	H26B—C26—H26E	141.1
C14—C13—C16	122.6 (2)	H26B—C26—H26F	56.3
C13—C14—H14	119.5	H26C—C26—H26D	56.3
C15—C14—C13	120.9 (2)	H26C—C26—H26E	56.3
C15—C14—H14	119.5	H26C—C26—H26F	141.1
N11—C15—C14	120.1 (2)	H26D—C26—H26E	109.5
N11—C15—H15	120.0	H26D—C26—H26F	109.5
C14—C15—H15	120.0	H26E—C26—H26F	109.5
C13—C16—H16A	109.5	Co1—O31—H31	121.0
C13—C16—H16B	109.5	C31—O31—Co1	123.18 (14)
C13—C16—H16C	109.5	C31—O31—H31	109.5
H16A—C16—H16B	109.5	O31—C31—H31A	109.5
H16A—C16—H16C	109.5	O31—C31—H31B	109.5
H16B—C16—H16C	109.5	O31—C31—H31C	109.5
Co1^i^—O21—Co1	107.47 (6)	H31A—C31—H31B	109.5
N21—O21—Co1^i^	130.26 (12)	H31A—C31—H31C	109.5
N21—O21—Co1	121.46 (12)	H31B—C31—H31C	109.5
O21—N21—C21	118.04 (18)		

Symmetry code: (i) −*x*+1, −*y*, −*z*+1.

### Hydrogen-bond geometry (Å, º)

**Table:**

*D*—H···*A*	*D*—H	H···*A*	*D*···*A*	*D*—H···*A*
C11—H11···N2	0.95	2.40	3.225 (3)	145
C12—H12···S1^ii^	0.95	2.79	3.688 (3)	158
C15—H15···S2^iii^	0.95	2.68	3.609 (3)	167
C21—H21···S2^iv^	0.95	3.03	3.917 (2)	156
C22—H22···S1^v^	0.95	2.98	3.821 (2)	148
O31—H31···S1^vi^	0.84	2.97	3.6106 (18)	134
O31—H31···O11^i^	0.84	2.31	3.003 (2)	141
C31—H31*B*···S2^iv^	0.98	2.83	3.575 (3)	133

Symmetry codes: (i) −*x*+1, −*y*, −*z*+1; (ii) −*x*+3/2, *y*+1/2, −*z*+3/2; (iii) *x*+1/2, −*y*+1/2, *z*−1/2; (iv) −*x*+1/2, *y*−1/2, −*z*+3/2; (v) *x*−1, *y*, *z*; (vi) −*x*+3/2, *y*−1/2, −*z*+3/2.
