# Supplementary figures and images for: Synthesis, crystal structure and thermal properties of the dinuclear complex bis­(μ-4-methylpyridine N-oxide-κ2 O:O)bis­[(methanol-κO)(4-methylpyridine N-oxide-κO)bis­(thio­cyanato-κN)cobalt(II)]
Source: Acta Crystallogr E Crystallogr Commun. 2024 Apr 18;80(Pt 5):481–5. doi: 10.1107/S2056989024003128 (PMC11074567; doi:10.1107/S2056989024003128)

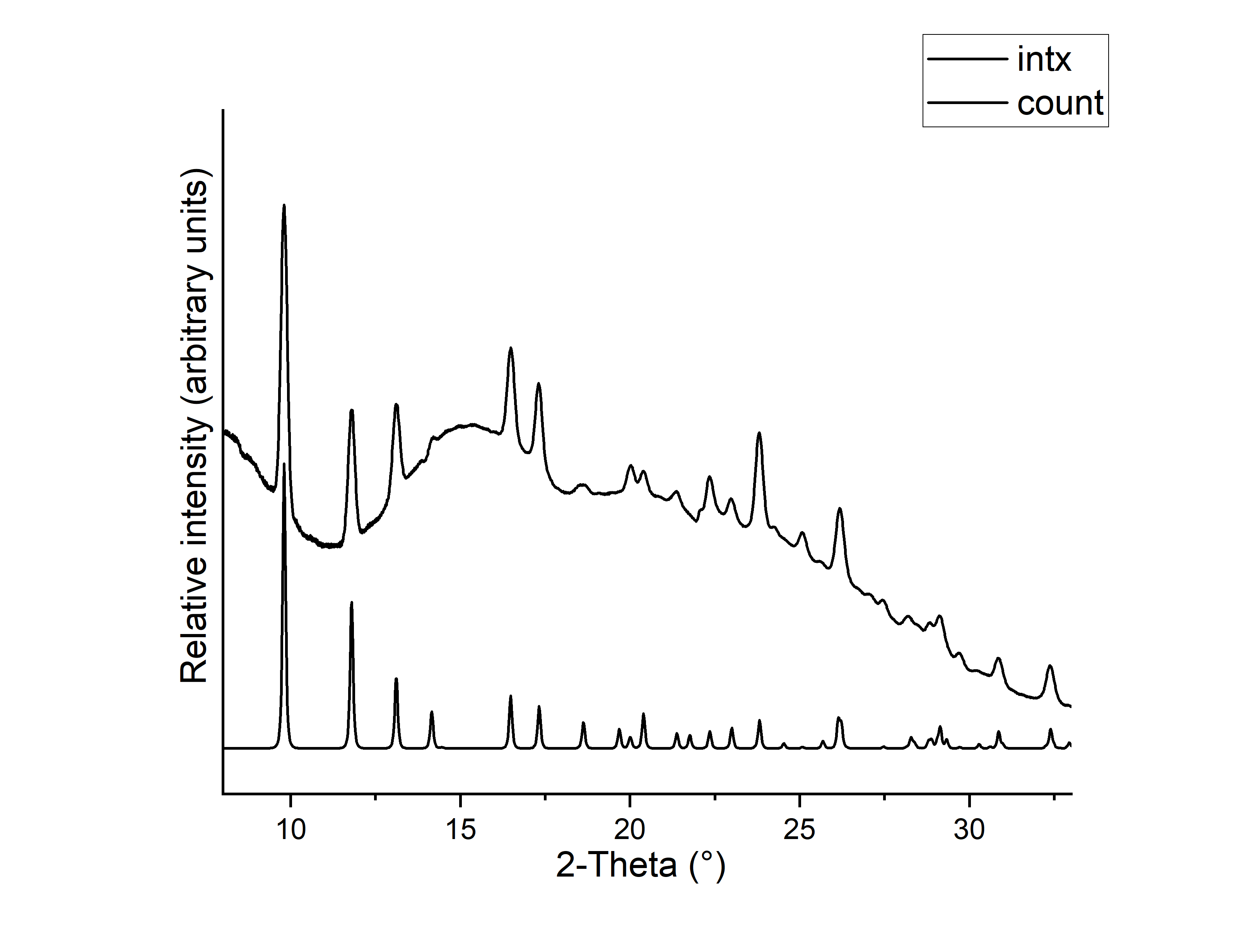

Supplement: Supplementary file 2 [file e-80-00481-sup3.png]
